# IO360° Speaker Interview: Q and A with Dr. Crystal Mackall

**DOI:** 10.4103/2666-2345.275505

**Published:** 2020-03-31

*The Journal of Immunotherapy and Precision Oncology is proud to support Immuno-Oncology360° and Cell & Gene Therapy Day.*

*Dr. Crystal Mackall is the Director of the Stanford Center for Cancer Cell Therapy at Stanford University School of Medicine and Director of the Parker Institute for Cancer Immunotherapy at Stanford. Prior to Stanford, Dr. Mackall spent 27 years with the National Cancer Institute and was a pioneer in understanding T-cell homeostasis and tumor immunology. She was among the first to demonstrate impressive activity of CD19-CAR for pediatric leukemia.*

*Dr. Mackall will discuss “New Scientific Frontiers in Cell Therapies for Solid Tumors” at **Cell & Gene Therapy Day** during the Immuno-Oncology360° event, February 26–28, 2020, in New York City. Dr Mackall recently did a Q and A with IO360° reporter Danny McCarthy. The highlights are below; for the full interview, visit http://www.io360summit.com/.*

**What inspired you to pursue medicine?** I always knew I wanted to be a physician, and when I started rotating on the wards, it was clear that cancer demanded the best of everything. The science of cancer – it's just always been the most interesting science out there. So, it was never a question as to what field I was going to go in. I just had trouble deciding if I was going to treat adults with cancer or children with cancer. I still feel that way about this field. If you can succeed here and make a difference, I just can't think of anything I'd rather spend my time doing.

**What about the field of immunotherapy is really exciting to you right now?** The most exciting thing right now is T-cell exhaustion. Our research has led us to conclude, especially as it pertains to CAR T-cells, that the problem of T-cell exhaustion is a particularly prevalent problem. So, we've been diving deep into that biology and have been able to unravel some new insights into the biology of T-cell exhaustion, and thereby engineer what we believe are exhaustion-resistant T-cells.

**Is there anything you'd like to see happen in the next few years?** It's very hard for us to run clinical trials that can efficiently look at many iterations. So, what I'm really hoping the field can do is evolve both the regulatory framework and the way we're delivering our vectors, so that we can be more nimble and we can get more patients treated more quickly, with more varied approaches.

**You're speaking at Cell & Gene Therapy Day in NYC about new frontiers in solid tumor immunotherapy. Can you tell us a little about that?** There are a lot of challenges to solid tumors. Some have to do with the target. But some of the time, it's simply the T-cells fail. And so, we really believe that unless you create a better offense, you make these cells stronger, more potent, more durable, that every kind of suppressive axis that the tumor throws at it is going to trip it up.

**Media Contact**

BreAnna Bugbee, Marketing Manager, The Conference Forum LLC, New York, NY, bre@tcfllc.org

